# Atypical skin manifestation in severe acute chikungunya infection in a pregnant woman: a case report

**DOI:** 10.1186/s13256-021-03197-3

**Published:** 2022-01-06

**Authors:** Saovanee Benjamanukul, Jira Chansaenroj, Chintana Chirathaworn, Yong Poovorawan

**Affiliations:** 1 Department of Medicine, Banphaeo General Hospital, Samutsakhon, 74120 Thailand; 2 Center of Excellence in Clinical Virology, Department of Pediatrics, Faculty of Medicine, Chulalongkorn University, Bangkok, 10330 Thailand; 3 Department of Microbiology, Faculty of Medicine, Chulalongkorn University, Bangkok, 10330 Thailand

**Keywords:** Skin manifestation, Atypical, Chikungunya, Pregnant woman

## Abstract

**Introduction:**

Patients with chikungunya virus infection commonly present with fever, skin rash, and severe joint pain. The vesiculobullous rash is rare in adults but common in infants. In addition, septic shock and acute respiratory distress syndrome are rare complications of atypical and severe acute chikungunya infection.

**Case presentation:**

We report the presence of an 18-year-old Thai female, at 31 weeks gestation, with fever, maculopapular rash, and polyarthritis. The rash later progressed to a vesiculobullous pattern, and she developed septic shock and acute respiratory distress syndrome. Skin biopsy and blood were positive for chikungunya virus RNA. The patient was intubated with a mechanical ventilator and subsequently fully recovered.

**Conclusion:**

Atypical skin manifestation and severe acute disease is likely due to immune response attenuation in pregnancy. The possibility of progression to severe or atypical disease in pregnant women suffering chikungunya should always be considered.

## Introduction

Chikungunya virus (CHIKV) is a viral illness transmitted by the *Aedes* mosquito. Acute clinical CHIKV infection is characterized by fever (typically > 38.5 °C) and arthralgia, which is usually incapacitating. The atypical disease is defined as laboratory-confirmed CHIKV associated with neurological, cardiovascular, dermatological, ophthalmological, hepatic, renal, respiratory, hematological, or other manifestations. Severe acute disease is defined as laboratory-confirmed CHIKV presenting with life-threatening dysfunction of at least one organ or system, requiring hospitalization [1]. Atypical and severe illness is more common in infants, the elderly, the immunocompromised, and pregnant patients [2, 3]. Septic shock and acute respiratory distress syndrome (ARDS) are rare complications of atypical and severe acute CHIKV disease [4–8]. Severe sepsis without septic shock was previously reported in pregnant patients infected with CHIKV [9]. Although vesiculobullous rash is common in infants, adults commonly manifest with a maculopapular rash [10]. Here, we report the development of septic shock and ARDS, and vesiculobullous rash manifestation in a pregnant patient.

## Case presentation

An otherwise healthy 18-year-old Thai female housewife at 31 weeks gestation, presented to our clinic complaining of fever and painful swollen wrists, hands, and feet for 6 hours prior to presentation. She could not make a fist and complained of difficulty walking. The patient complained of a headache but denied suffering nausea, vomiting, diarrhea, or myalgia. A pruritic, erythematous rash on her abdomen was noted. She denied recent hospitalization and was not taking any medications. No family members had similar conditions. The patient stated that several of her neighbors also suffered fever and arthralgia within the past week. Her vital signs were as follows: body temperature, 38.1 °C; blood pressure, 100/40 mmHg; pulse rate, 126 beats/min; respiratory rate, 20 breaths/min; and O_2_ saturation of 98% on room air. She was hospitalized due to suspected septic shock.

**Fig. 1:**
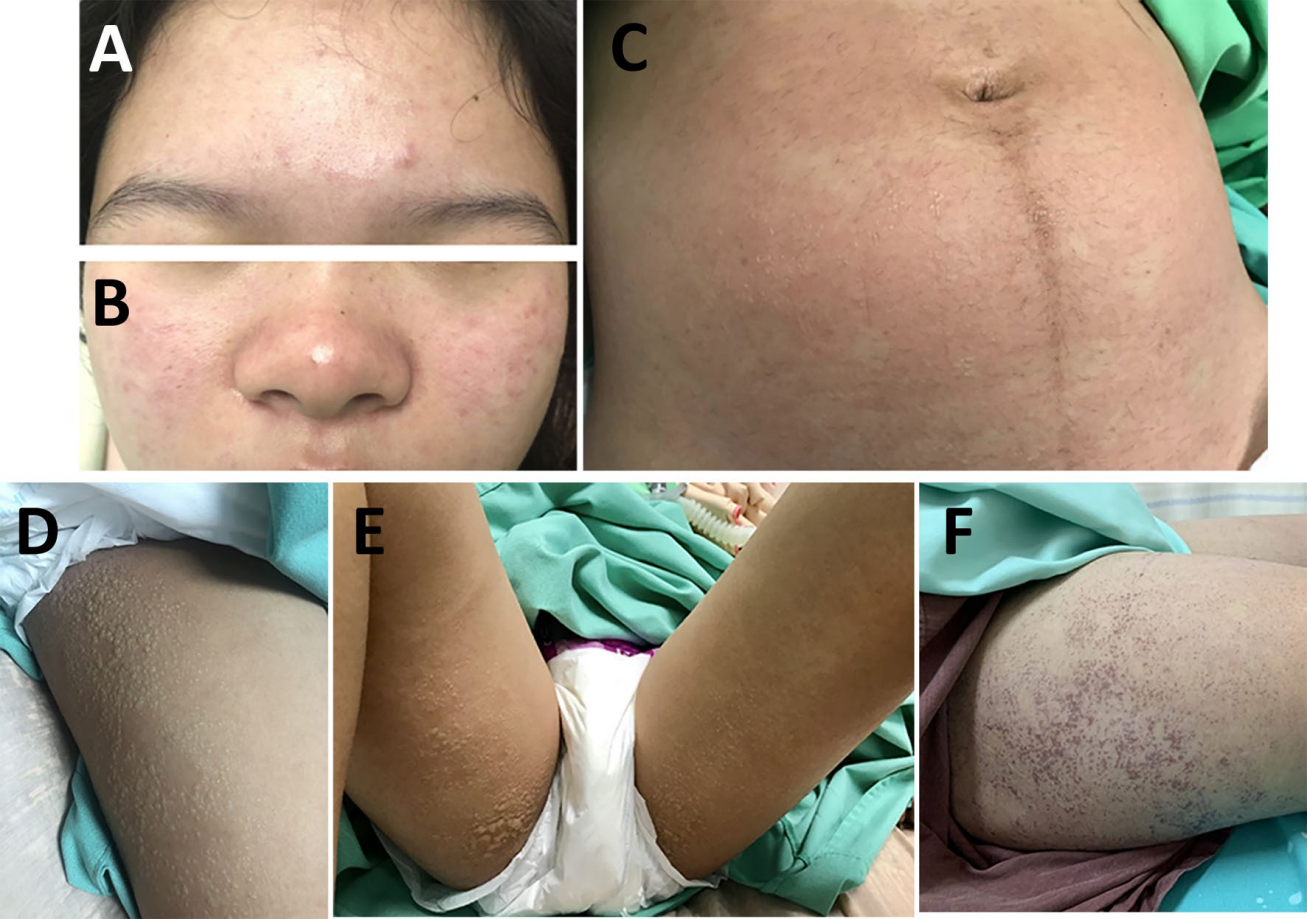
Skin manifestations revealed **A** maculopapular rashes on the forehead **B** cheeks and nose, as well as **C**–**E** multiple small, clear vesicles on an erythematous maculopapular background on the abdomen. A nonhemorrhagic vesicular rash on the lateral side of both thighs with some bullae formation on the ventral side of the upper thighs was also noted. **F** Numerous discrete, crusting, and coalescing hemorrhagic vesicles on the right thigh were noted

On examination at admission, the patient had maculopapular rashes on the cheeks, nose, forehead, and abdominal wall (Fig. 1A). Cervical lymph nodes were not palpable. Her heart and lung examinations were unremarkable. The uterus was palpated at three fingers above the umbilicus. Exam of her extremities revealed arthritis of both wrists and ankles as well as all metatarsophalangeal joints. Arthrocentesis of the left wrist yielded clear yellow synovial fluid with high viscosity (0.1 CCS). Gram staining did not reveal the presence of organisms. Complete blood count revealed a hemoglobin count of 10.8 g/dL, hematocrit of 33%, white blood cell count of 9300 cells/mm^3^ (neutrophils, 91%; leukocytes, 5%; eosinophils, 0%; monocytes, 4%; basophils, 0%), and platelet count of 180,000/mm^3^. Blood urea nitrogen and creatinine were found to be 8.3 mg/dL and 0.38 mg/dL, respectively. Liver function testing revealed total protein of 6.2 g/dL, albumin of 3.5 g/dL, globulin of 2.7 g/dL, total bilirubin of 0.4 mg/dL, and direct bilirubin of 0.1 mg/dL. Levels of aspartate transaminase (AST), alanine transaminase (ALT), and alkaline phosphatase (ALP) were 15 U/L, 8 U/L, and 102 U/L, respectively; erythrocyte sedimentation rate (ESR) was 52 mm/hour.

**Fig. 2:**
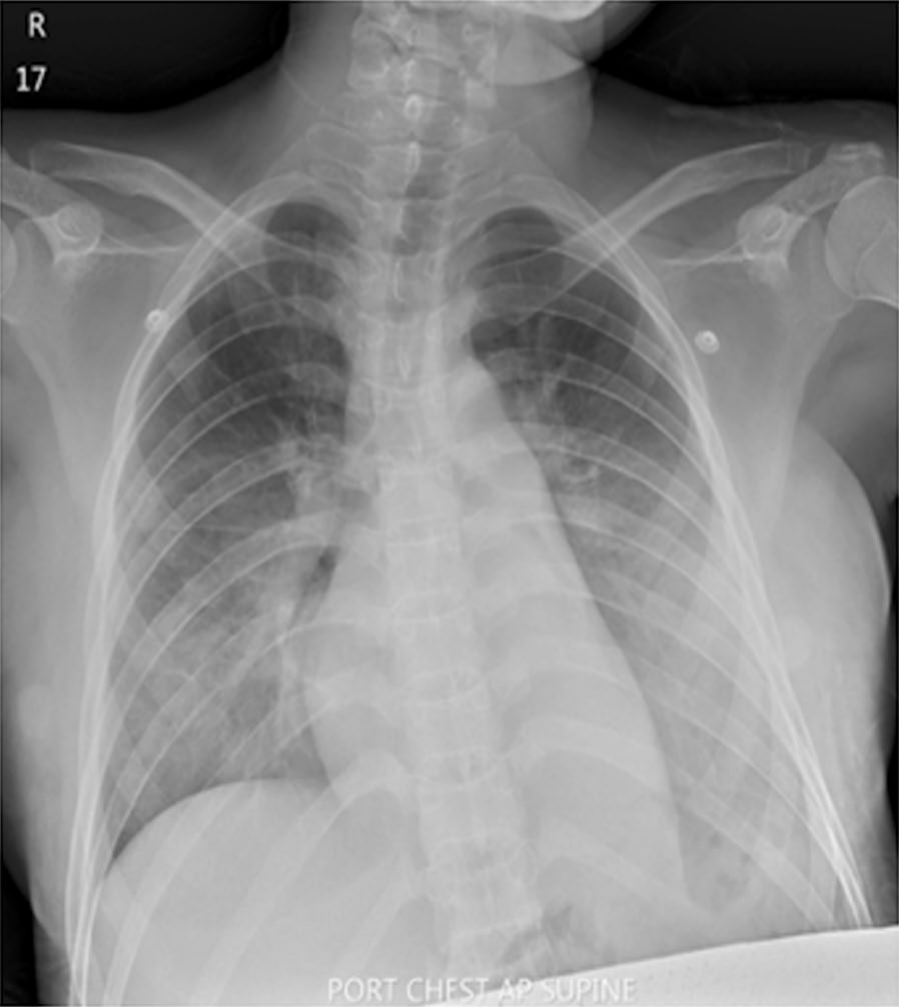
Chest X-ray revealed airspace opacifications with bronchogram presence in the perihilar and lower lung areas bilaterally. No cardiomegaly was demonstrated

Serology for dengue nonstructural protein 1 (NS1) antigen, immunoglobulin M (IgM), and immunoglobulin G (IgG) was negative. The patient was initially diagnosed with viral arthritis, but septic arthritis could not be ruled out. Thus, she was treated with intravenous ceftriaxone 2 g once daily on the first day of admission, and her hypotension was managed with intravenous fluid loading. On the second day of admission, her arthralgia improved greatly, but she still had a high-grade fever and worsening hypotension. Ceftriaxone was subsequently switched to intravenous meropenem 1 g every 8 hours. The patient also developed hypoxemia while administering oxygen via a nonrebreather mask at 10 L/minute. On auscultation, her lungs were clear; a chest X-ray revealed no abnormalities. Imaging by computed tomography (CT) angiography showed no evidence of pulmonary embolism. Bilateral pulmonary consolidations at the superior–lateral–posterior basal segments associated with diffuse ground-glass infiltration and bilateral pleural effusions with a fluid-filled right minor fissure were noted. Sinus tachycardia was reported on electrocardiography. On the third day of admission, noradrenaline was administered to manage worsening hypotension. Echocardiography revealed good ventricular systolic contraction, no regional wall motion abnormality, and no pericardial effusion. The inferior vena cava was 1.8 cm in diameter with an inspiratory collapse of < 50%. We initially considered the diagnosis of noncardiogenic pulmonary edema; differential diagnoses included viral pneumonia and tropical infection. Thus, azithromycin 500 mg intravenous once daily and oseltamivir 75 mg twice daily were added to her treatment regimen. The patient suffered progressive tachypnea and hypoxemia (O_2_ saturation 93–95%) on high-flow oxygen. A further chest X-ray revealed airspace opacifications with bronchogram presence in the perihilar and lower lung areas bilaterally. No cardiomegaly was demonstrated (Fig. 2), and she was intubated on a mechanical ventilator. As the PaO_2_:FiO_2_ ratio was less than 300, ARDS was diagnosed. A vesicular rash was found along both thighs, legs, and abdomen with bullae formation on the perineum and buttocks (Fig. 1B–D). Tzanck smear did not reveal the presence of multinucleated giant cells. After 3 days of invasive ventilation, the patient was extubated. No adverse or unanticipated events occurred. Body temperature returned to normal baseline levels, and she fully recovered from arthralgia within the next 3 days. The skin lesion on the abdomen and both thighs became crusted hemorrhagic vesicles 6 days later (Fig. 1E), and return of normal skin color was observed on follow-up. She proceeded to deliver a healthy infant at full term.

**Fig. 3:**
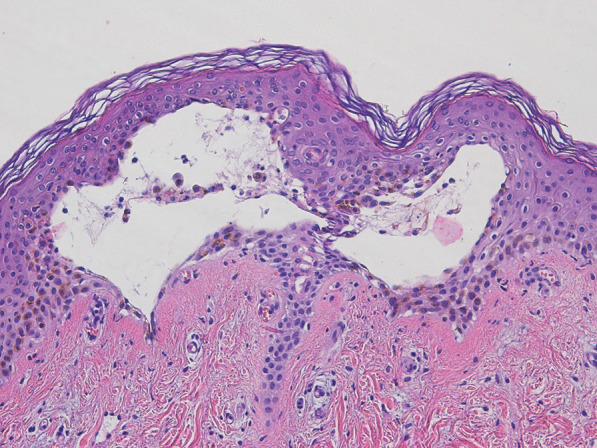
Skin biopsy revealed subepidermal vesicles containing acantholytic cells and reepithelization in the epidermis

Additional laboratory tests were performed during the patient’s time in the hospital. A positive polymerase chain reaction (PCR) for CHIKV RNA was reported 1 week after the admission date because CHIKV PCR could not be accessed in the secondary care hospital. Skin biopsy revealed subepidermal vesicles containing acantholytic cells and reepithelization in the epidermis. Superficial and deep perivascular infiltration with lymphocytes in the dermis was noted (Fig. 3).

**Fig. 4:**
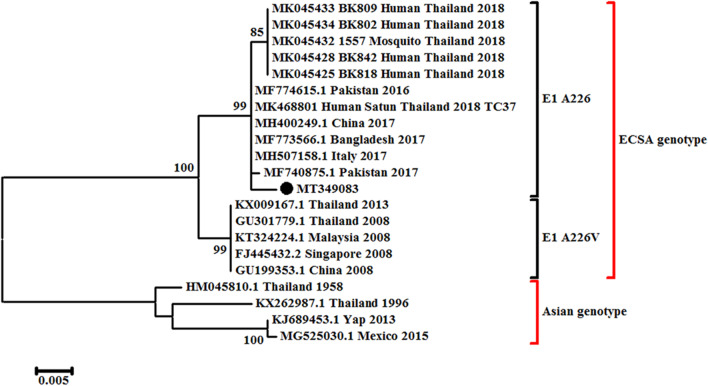
Phylogenetic analysis of the chikungunya E1 gene from patient samples and comparison with reference strains. The scale bar represents nucleotide substitutions per site. Strains are shown with accession numbers and classified according to country and sampling year. The black dot indicates the strain identified as infecting our patient.

Skin biopsy analysis revealed CHIKV RNA, and phylogenetic analysis revealed that the virus belonged to the ECSA genotype (A226V E1 protein mutation; Fig. 4). Blood and urine PCR for Zika were negative. Scrub and murine typhus IgM and IgG were also negative. Testing for human immunodeficiency virus (HIV) was negative. Antinuclear antibody staining was 1:40 (homogeneous and finely speckled).

## Discussion

The CHIKV is transmitted to humans via mosquito vectors. The virus has been reported to infect macrophages, fibroblasts, and endothelial cells in the skin. The CHIKV may enter lymph nodes and disseminate to various tissues such as muscle, joint, liver, and brain [11, 12]. Patients suffering CHIKV infection typically present with fever, skin rash, and incapacitating arthralgia. Cutaneous manifestation of CHIKV in adults varies according to location and appearance; most are typified by a maculopapular rash [13]. Formation of bullae and skin blistering, common in infants less than 6 months of age, is rare in adults [10, 14, 15]. Real-time PCR for CHIKV RNA in blister fluid was positive and yielded a mean viral load higher than concurrent serum. Vesicles likely result from viral replication in the epidermis, causing focal necrosis, ballooning degeneration, or nuclear disruption followed by an immune response and subsequent leukocyte infiltration. Pediatric patients appear to exhibit severe dermatological manifestations compared with the adult population [16, 17]. Some adults with alcoholism were reported to suffer extensive hemorrhagic bullous lesions on the upper and lower limbs [18].

Shock and ARDS are rare complications of CHIKV sepsis. Our patient suffered a severe acute CHIKV infection and manifested with an atypical presentation. We defined the acute phase of illness according to the definition provided by the World Health Organization [19]. During the 2005–2006 Le Reunion outbreak, the severe disease occurred in only 0.3% of symptomatic patients, and 36% of atypical cases were considered critical with an estimated case fatality rate of 1 in 1000 [6]. Severe disease was reported in 5.2% of patients from the French Guiana outbreak. Septic shock is rarely reported in CHIKV [3, 4, 20]. To date, no study has reported CHIKV infection to be more severe in pregnancy than in nonpregnant women [8, 9, 21].

Our patient had an illness consistent with the case definition for septic shock [22]. Laboratory tests suggested that this illness resulted from CHIKV infection. Bacterial and dengue infections, common etiologies of tropical diseases, were ruled out. The virus can cause sepsis and microvascular leakage either directly through cytopathic effects or indirectly by inducing systemic cytokinemia and dysregulation of endothelial barriers, leading to increased vascular permeability and immune cell migration into tissues. Moreover, an aberrant immune response in the setting of viral sepsis has been reported to fail to clear viral infection [23–25]. The severity of viral sepsis, greater in immunocompromised patients, likely depends on viral virulence. Newborns, infants, and the elderly are predisposed to suffer more severe diseases [6, 26].

Our patient was in the third trimester of pregnancy. Relative immune suppression and susceptibility to infection are commonly found in pregnant women. Differences in cytokines and hormonal shifts in each trimester affect sensitivity to different infectious diseases [27–29]. Immunologic alterations in the setting of advancing pregnancy thus impair pathogen clearance, resulting in increased severity of diseases such as influenza, malaria, and herpes simplex virus infection [29, 30]. Prior research has reported that 15% of pregnant women required treatment in the intensive care unit; of these patients, 89% were in the last trimester of pregnancy. Most of these patients (78%) met diagnostic criteria for severe sepsis, but none suffered septic shock. Respiratory dysfunction was not found to affect morbidity in severe acute disease [9].

Sepsis-induced ARDS is initiated by an inflammatory host response to microbial agents. Subsequent dysregulation of this response drives vascular barrier disruption. Respiratory failure and ARDS due to CHIKV infection have rarely been reported; some studies reported only respiratory failure but did not identify the etiology of respiratory dysfunction in severe or atypical cases [3, 4, 6, 7, 20, 31]. Only one case report describing ARDS in a nonpregnant patient has been published to date [32].

The limitation of this case report was the absence of the PCR for influenza, which also causes ARDS [33].

## Conclusion

Our 18-year-old pregnant patient suffering from CHIKV infection manifested with atypical characteristics such as vesiculobullous lesions and ARDS. Atypical skin manifestations likely resulted from attenuation of the immune response in the setting of pregnancy. Replication of CHIKV in skin cells facilitates high levels of viral particle entry into the bloodstream, subsequently resulting in ARDS. Unfortunately, viral load and cytokine levels could not be investigated in this patient. This case report underscores the importance of awareness of the possibility of CHIKV progression to severe disease and manifestation with atypical skin lesions in pregnant women.

## Data Availability

All relevant data are contained in the article. There are no supplementary data.

## Abbreviations

ARDS: Acute respiratory distress syndrome
CHIKV: Chikungunya virus
PCR: Polymerase chain reaction
ECSA: East–Central–South–African
HSV: Herpes simplex virus
AST: Aspartate aminotransferase
ALT: Alanine aminotransferase
ALP: Alkaline phosphatase
ESR: Erythrocyte sedimentation rate
NS1Ag: Nonstructural protein 1 antigen
IgM: Immunoglobulin M
IgG: Immunoglobulin G
PaO_2_/FiO_2_: Ratio of arterial oxygen partial pressure to the fraction of inspired oxygen
